# NAD^+^ regulates Treg cell fate and promotes allograft survival via a systemic IL-10 production that is CD4^+^ CD25^+^ Foxp3^+^ T cells independent

**DOI:** 10.1038/srep22325

**Published:** 2016-03-01

**Authors:** Abdallah Elkhal, Hector Rodriguez Cetina Biefer, Timm Heinbokel, Hirofumi Uehara, Markus Quante, Midas Seyda, Jeroen M. Schuitenmaker, Felix Krenzien, Virginia Camacho, Miguel A. de la Fuente, Ionita Ghiran, Stefan G. Tullius

**Affiliations:** 1 Division of Transplant Surgery and Transplantation Surgery Research Laboratory, Brigham and Women’s Hospital, Harvard Medical School, Boston-02115, MA, USA; 2 Clinic for Cardiovascular Surgery, University Hospital Zurich, Zurich-8006, Switzerland; 3 Institute of Medical Immunology, Charité – Universitätsmedizin Berlin, Berlin-10117, Germany; 4 Flow Cytometry Core Facility, Beth Israel Deaconess Medical Center, Harvard Stem Cell Institute, Boston, Massachusetts 02115, USA; 5 Instituto de Biología y Genética Molecular, University of Valladolid, Valladolid-47003, Spain; 6 Department of Medicine, Beth Israel Deaconess Medical Center, Harvard Medical School, Boston, Ma. 02115, USA

## Abstract

CD4^+^ CD25^+^ Foxp3^+^ Tregs have been shown to play a central role in immune homeostasis while preventing from fatal inflammatory responses, while Th17 cells have traditionally been recognized as pro-inflammatory mediators implicated in a myriad of diseases. Studies have shown the potential of Tregs to convert into Th17 cells, and Th17 cells into Tregs. Increasing evidence have pointed out CD25 as a key molecule during this transdifferentiation process, however molecules that allow such development remain unknown. Here, we investigated the impact of NAD^+^ on the fate of CD4^+^ CD25^+^ Foxp3^+^ Tregs in-depth, dissected their transcriptional signature profile and explored mechanisms underlying their conversion into IL-17A producing cells. Our results demonstrate that NAD^+^ promotes Treg conversion into Th17 cells *in vitro* and *in vivo* via CD25 cell surface marker. Despite the reduced number of Tregs, known to promote homeostasis, and an increased number of pro-inflammatory Th17 cells, NAD^+^ was able to promote an impressive allograft survival through a robust systemic IL-10 production that was CD4^+^ CD25^+^ Foxp3^+^ independent. Collectively, our study unravels a novel immunoregulatory mechanism of NAD^+^ that regulates Tregs fate while promoting allograft survival that may have clinical applications in alloimmunity and in a wide spectrum of inflammatory conditions.

CD4^+^ CD25^+^ Foxp3^+^ natural regulatory T cells (nTregs) play a critical role in the maintenance of immune tolerance and T cell homeostasis in mouse and human^1^,^2^. It is well established that Tregs inhibit autoimmunity and inflammation through multiple mechanisms including the production of IL-10. Alternative mechanisms may work through TGF-β, known to suppress IFNγ and T-bet expression, a master regulator of T helper 1 (Th1) cells^3^. Tregs, were first described by Sakaguchi and co-workers^4^ and have since been recognized as a CD4^+^ T cell type in both, mice and humans, characterized as CD4^+^ CD25^+^ Foxp3^+^ Tregs constituting a distinct thymus-derived T cell lineage. An additional type of Tregs has been characterized and termed induced regulatory T cells (iTregs). These cells originate in the periphery upon T cell receptor (TCR) stimulation in the presence of TGF-β^2^ as shown in mouse studies. Although many studies have characterized particularly nTregs as a stable lineage, recent observations in mice have challenged this concept^5^,^6^. It has been shown that CD4^+^ CD25^+^ Foxp3^+^ cells are comprised of stable (CD4^+^ CD25^high^Foxp3^+^) and unstable (CD4^+^ CD25^low^Foxp3^+^) populations linked to the expression of the cell surface marker CD25^7^,^8^. An additional type of Tregs, termed regulatory type 1 (Tr1) cells, has recently been reported in mouse and human experiments^9^. Tr1 cells have been shown to have the capacity to co-produce IFNγ and IL-10^10^. It is well established that IFNγ-producing cells that co-express IL-10 have immunoregulatory properties that have the capacity to inhibit inflammation, promote transplant tolerance and prevent tissue damage^11^. More importantly, very recently it has been reported that pro-inflammatory Th17 cells can convert into immunoregulatory Tr1 cells in mice^12^.

Furthermore, increasing evidences point towards the existence of CD4^+^ T cells that co-express IL-17A and Foxp3^10^,^13^,^14^,^15^. A recent study has shown the importance of CD25 expression levels for the differentiation of CD4^+^ CD25^+^ Foxp3^+^ Tregs into Th17 cells^11^. Moreover, it has been recently shown that nicotinamide adenine dinucleotide (NAD^+^), a natural co-factor has the ability to modify the binding of IL-2 to CD25^16^. The role of NAD^+^ and CD25 in Tregs fate remains however unknown.

Here, we investigated the impact of NAD^+^ on the fate of Tregs. In detail, we characterized the impact of NAD^+^ on the stability of CD25 while testing the impact on Th17 differentiation. Our study demonstrates that NAD^+^ favors the conversion of CD4^+^ CD25^+^ Foxp3^+^ Tregs into IL-17A producing cells through purinergic signaling that involves the transcription factor STAT-3. Moreover, NAD^+^ resulted in a selective depletion of murine CD4^+^ CD25^High^Foxp3^+^ Tregs that was associated with a transdifferentiation of CD4^+^ CD25^Low^Foxp3^+^ Tregs into IL-17A producing cells exhibiting Th17 cells transcriptional and cytokine profiles. In summary, our study underscores a robust and unique immunoregulatory property of NAD^+^ with broad anti-inflammatory and immunosuppressive capacities with a wide spectrum of potential clinical applications.

## Results

### NAD^+^ promotes Treg conversion into Th17 cells and their proliferation *in vitro* in absence of TGF-β, IL-6, IL-23 and in presence of IL-2

Recent reports have challenged the notion that Tregs represent a stable lineage^17^. It has been proposed that Tregs may lose Foxp3 expression under specific inflammatory conditions, thus acquiring effector functions^17^,^18^. In addition, several studies have shown that Tregs can convert into Th17 cells^10^,^19^,^20^. More recently, a study demonstrated that Th17 can convert into regulatory T cells^12^. Furthermore, TGF-β, IL-6, IL-21 have been shown to be critical for Th17 differentiation subsequent to TCR engagement while IL-23 promoted Th17 proliferation^21^. In contrast, addition of IL-2 has been shown to prevent Th17 differentiation^22^,^23^. We have previously shown that NAD^+^ promotes CD4^+^ IL17A^+^ T cells *in vivo* and *in vitro*. More importantly, we have shown that NAD^+^ regulated the fate of CD4^+^ T cells and in particular that of CD4^+^ CD25^+^ Foxp3^+^ 24. Accumulating evidences suggest that CD4^+^ CD25^+^ Foxp3^+^ cells are comprised of stable and unstable populations characterized by their expression of CD25^7^,^8^. Of particular relevance, mechanisms by which NAD^+^ can alter Tregs remain unclear. Moreover, it is unknown whether CD25 plays a role in this process. Thus, we dissected the impact of NAD^+^ on Tregs and evaluated the role of CD25 on the fate of Tregs.

**Figure 1:**
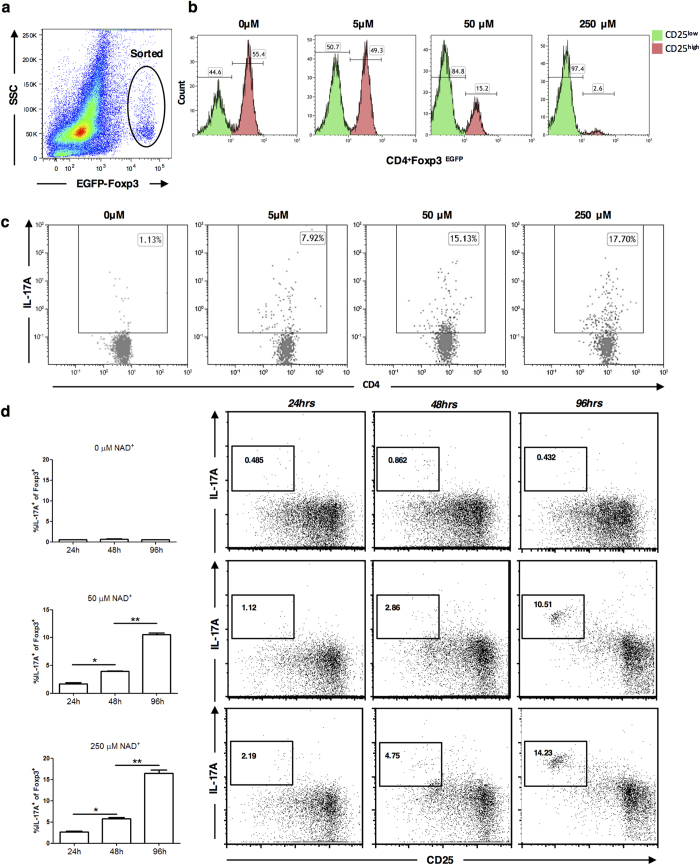
NAD^+^ promotes conversion of CD4^+^ CD25^+^ Foxp3^+^ Tregs into IL-17A producing cells. (**a**) CD4^+^ CD25^+^ Tregs were FACS-sorted from EGFP-Foxp3 mice and cultured in presence of α-CD3, α-CD28, and IL-2 with increasing concentrations of NAD^+^ for 96h. (**b**) Frequencies of CD4^+^ CD25^High^Foxp3^EGFP^ and CD4^+^ CD25^Low^Foxp3^EGFP^ T cells were assessed in a dose-dependent manner. (**c**) CD4^+^ CD25^+^ Tregs from EGFP-Foxp3 mice were isolated and frequencies of CD4^+^ Foxp3^+^ IL-17A^+^ cells were assessed in a dose-dependent manner for 96h (*n* = 12 per group, representative plots shown). (**d**) CD4^+^ CD25^+^ Tregs from wild type mice were isolated and frequencies of CD4^+^ Foxp3^+^ IL-17A^+^ cells were assessed in a dose and time-dependent manner (*n* = 12 per group, representative plots shown). Data derived from three independent experiments. Data represent mean ± s.d. *P < 0.05; **P < 0.01. Student’s *t*-test and ANOVA tests were used accordingly to compare groups.

CD4^+^ CD25^+^ Foxp3^+^ Tregs were sorted from EGFP-Foxp3 mice (Fig. 1a) and cultured for a period of 96 hours in presence of increasing NAD^+^ concentrations and TCR activation. Our flow cytometry data indicated increased frequencies of CD4^+^ CD25^Low^Foxp3^EGFP^ but not CD4^+^ CD25^High^Foxp3^EGFP^ T cells (Fig. 1b). Of note, only CD4^+^ CD25^Low^Foxp3^EGFP^ cells were able to produce IL-17A in presence of NAD^+^ in a dose dependent manner (Fig. 1c). Similar results were observed with isolated CD4^+^ CD25^+^ T cells from wild type mice (purity > 98%, Supplementary Fig. S1a), cultured at different time points under varying concentrations of NAD^+^ (Fig. 1d). Of note, CD4^+^ CD25^+^ EGFP^+^ Foxp3^+^ cells were cultured without TGF-β, IL-6 and in presence of IL-2 (50 ng/ml). Interestingly, NAD^+^ concentrations were linked to reduced frequencies of CD4^+^ CD25^High^Foxp3^+^ but not CD4^+^ CD25^Low^Foxp3^EGFP^ T cells (Fig. 1b and Supplementary Fig. S1b) as a result of apoptosis (Supplementary Fig. S1c). More importantly, in parallel to the reduced frequencies of CD4^+^ CD25^High^Foxp3^+^ T cells, our results indicated an increased frequency of CD4^+^ CD25^Low^Foxp3^+^ IL-17A^+^ cells (Supplementary Fig. S2). Indeed, after 96 hrs of culture in the presence of 250 μM of NAD^+^ more than 14% of isolated CD4^+^ CD25^Low^Foxp3^+^ T cells from EGFP-Foxp3 (Fig. 1c) or wild type mice (Fig. 1d) produced IL-17A.

Next, we tested the mechanisms leading to increasing numbers of CD4^+^ CD25^Low^IL-17A^+^ Foxp3^+^ cells. Isolated Tregs were cultured in the presence of increasing concentrations of NAD^+^ and apoptosis and proliferation of CD4^+^ CD25^Low^IL-17A^+^ Foxp3^+^ cells were assessed after 48 and 96 hrs. Our results showed not only an absence of apoptosis but also a dose dependent proliferation of CD4^+^ CD25^Low^IL-17A^+^ Foxp3^+^ in presence of NAD^+^ (Supplementary Fig. S2a and S2b). Taken together, these results demonstrate that NAD^+^ induces the conversion of CD4^+^ CD25^+^ EGFP^+^ Foxp3^+^ into CD4^+^ IL-17A producing T cells in the absence of exogenous TGF-β, IL-6 and IL-23, cytokines previously considered necessary for Th17 activation, proliferation and maintenance^21^, and in the presence of IL-2, a Th17 inhibitory cytokine^25^.

### NAD^+^ specifically promotes the conversion of Treg into Th17 cells

Next, we investigated whether NAD^+^ was able to specifically promote Treg conversion into Th17 cells by assessing transcriptional and cytokine profiles of CD4^+^ CD25^+^ Foxp3^+^ that differentiated into CD4^+^ IL-17A^+^ cells. Therefore, we measured mRNA and cytokine levels that are specific to Th1, Th2, Tregs and Th17 by real-time PCR and ELISA produced by isolated Tregs in presence of increasing NAD^+^ concentrations at different time points.

**Figure 2:**
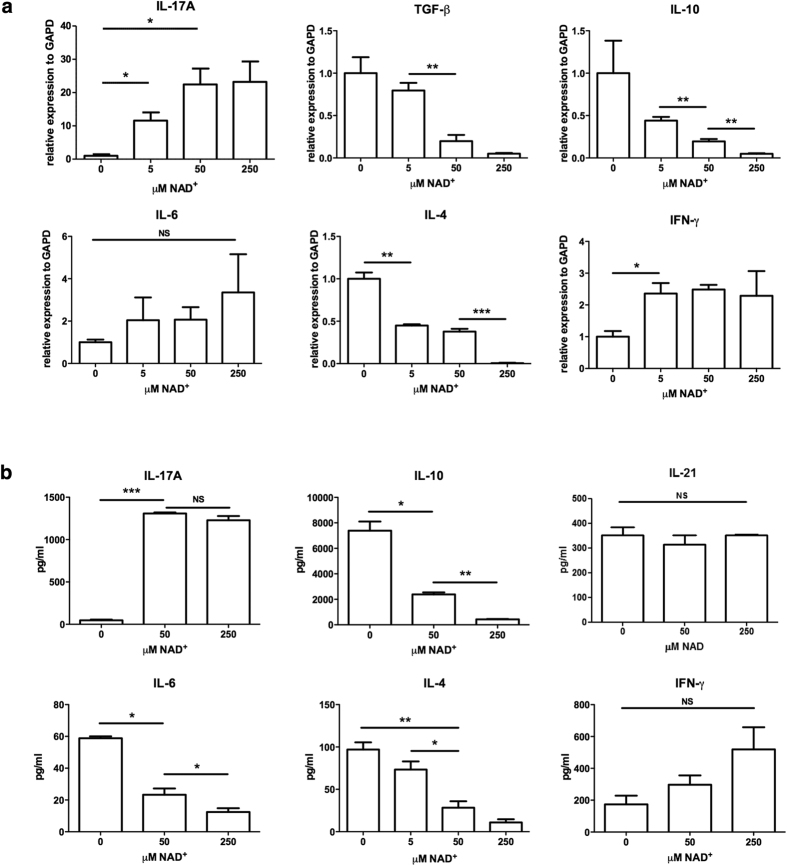
NAD^+^ converts Tregs into Th17 cells specifically. CD4^+^ CD25^+^ Tregs were cultured in presence of α-CD3, α-CD28, and IL-2 with increasing concentrations of NAD^+^ and after 96 hrs (**a**) mRNA and cytokine levels of Treg (*TGF-β*, *IL-10*), Th1 (*IFNγ*) Th2 (*IL-4*, *IL-6*, *IL-10*) and Th17 (*IL-17A*) cytokines were measured/quantified by real-time PCR (*n* = 8 per group) or (**b**) ELISA (*n* = 8 per group) respectively. Data derived from two independent experiments. Data represent mean ± s.d.*P < 0.05; **P < 0.01; ***P < 0.001; NS, not significant. ANOVA tests were used to compare groups.

When CD4^+^ CD25^+^ Foxp3^+^ were cultured in presence of NAD^+^, IL-17A mRNA (Fig. 2a) and protein levels (Fig. 2b) increased in a dose dependent manner. At the same time, mRNA of IL-10 and TGF-β and cytokine levels of IL-10, cytokine typically produced by Tregs decreased dramatically (Fig. 2a,b). Similarly, mRNA and protein levels of IL-4, a cytokine typically produced by Th2 cells, decreased in a dose dependent manner in the presence of NAD^+^ (Fig. 2a,b). Of note, we observed a minor NAD^+^ dose independent increase of IFNγ, a typical Th1 cytokine. In addition, protein levels of IL-21 and IL-23, two cytokines that are known to be essential for Th17 development, were measured. We observed only modest levels of IL-21 cytokine, which remained unchanged with increasing doses of NAD^+^ , while IL-23 could not be detected. Collectively, our results indicated that NAD^+^ promoted specifically the expression and secretion of IL-17A cytokine.

**Figure 3:**
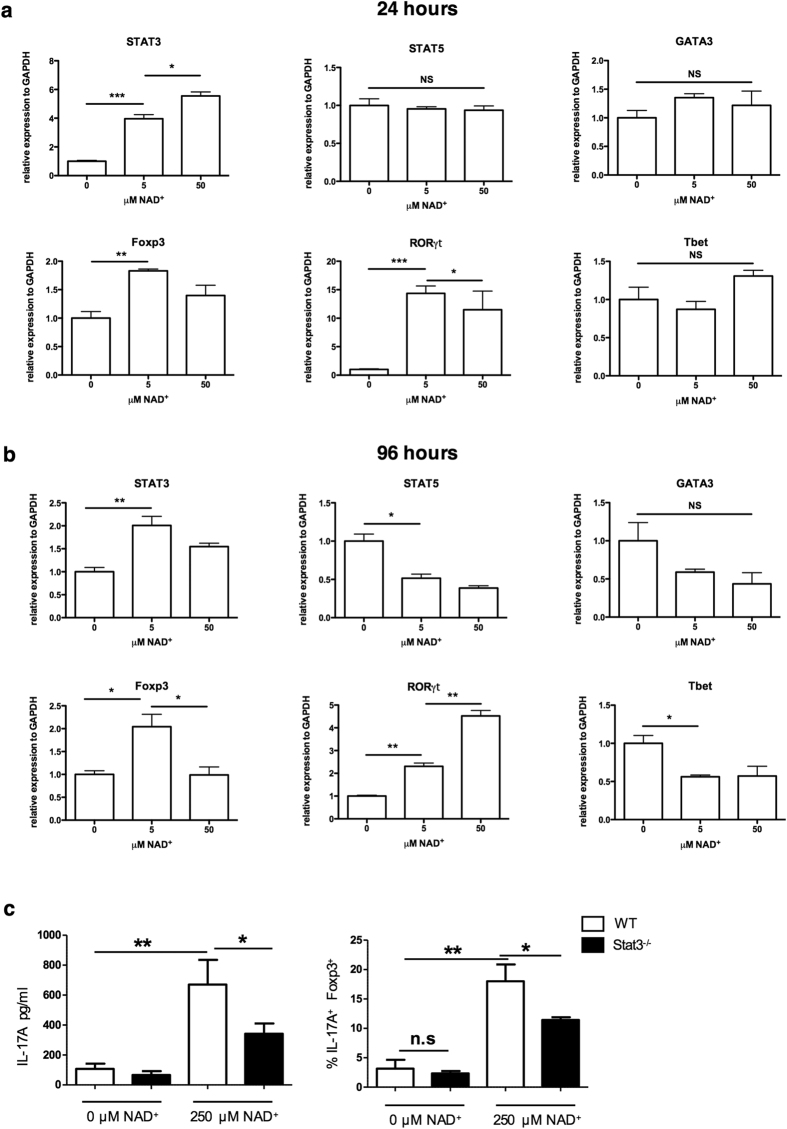
NAD^+^ promotes Treg conversion into Th17 cells through the transcription factors STAT3 and RORγt. CD4^+^ CD25^+^ Tregs were cultured in presence of α-CD3, α-CD28, and IL-2 with increasing concentrations of NAD^+^; by (**a**) 24 hrs or (**b**) 96 hrs of culture mRNA levels of *Tbx21* (*Tbe*t), *GATA3*, *STAT3*, *STAT5*, *RORγt* and *Foxp3* were measured by real-time PCR (*n* = 6 per group), NAD^+^ was added at increasingly concentreations of 0, 5 and 50 μM. (**c**) CD4^+^ CD25^+^ Tregs isolated from *STAT3*^−/−^ mice were cultured for 96 hrs in the presence of α-CD3, α-CD28, and IL-2 with or without NAD^+^ 250 μM ; frequencies of CD4^+^ IL-17A^+^ Foxp3^+^ cells and IL-17A protein levels were determined by flow cytometry and ELISA (*n* = 6 per group). Data derived from two independent experiments. Data represent mean ± s.d. *P < 0.05; **P < 0.01; ***P < 0.001; NS, not significant. ANOVA tests were used to compare groups.

It is known that both the cytokine environment and the presence of transcription factors are the major drivers for CD4^+^ T cell differentiation^26^. Thus, we next assessed the impact of NAD^+^ on the signature transcriptional profile of Th17 and Tregs cells in particular STAT3 and RORγt, two transcription factors that are essential for Th17 differentiation, and Foxp3, a transcription factor required for Treg differentiation and maintenance. To evaluate whether NAD^+^ induced a change in the signature transcriptional profile, isolated Tregs were cultured in presence of increasing NAD^+^ concentrations and mRNA levels for *Tbx21* (also known as *Tbet*), *GATA3*, *Foxp3*, *STAT3* and *STAT5* were measured by 24 hrs and 96 hrs, respectively (Fig. 3a,b). After 24 hrs of culture, *STAT3* (>5 fold) and *ROR*γ*t* (>10 fold) were significantly increased, while *Foxp3* showed only a modestly increase. Of note, the expression levels of *Tbx21*, *GATA3*, *Foxp3* and *STAT5* remained unchanged (Fig. 3a). The increase levels of *STAT3* and *ROR*γ*t* remained significantly increased after 96 hrs of culture while mRNA levels of *Tbx21*, *GATA-3*, and *STAT5* had decreased significantly (Fig. 3b).

It has been shown that *STAT3* can attenuate *Foxp3* expression while promoting Th17 development^27^. Our results indicated that NAD^+^ enhanced *STAT3* expression levels. Thus, we tested whether NAD^+^ promotes the conversion of Treg into Th17 cells through the transcription factor STAT3. Tregs were isolated from *STAT3*^−/−^ and wild type (WT) mice and cultured with or without NAD^+^. CD4^+^ IL-17A^+^ cells and IL-17A cytokine production were quantified after 96 hrs by flow cytometry and ELISA, respectively. Our results indicated that Tregs from *STAT3*^−/−^ mice had a significantly decreased frequency of IL-17A^+^ cells with a reduced IL-17A production (Fig. 3c) suggesting that NAD^+^ induces the conversion of Tregs into Th17 cells, at least in part, through the transcription factor STAT3.

Thus, our results indicate that CD4^+^ CD25^+^ Foxp3^+^ Tregs lose their cytokine and transcriptional profile in the presence of NAD^+^, while acquiring a Th17 cytokine and transcriptional signature.

### NAD^+^ promotes the conversion of Tregs into Th17 cells through purinergic receptors

**Figure 4:**
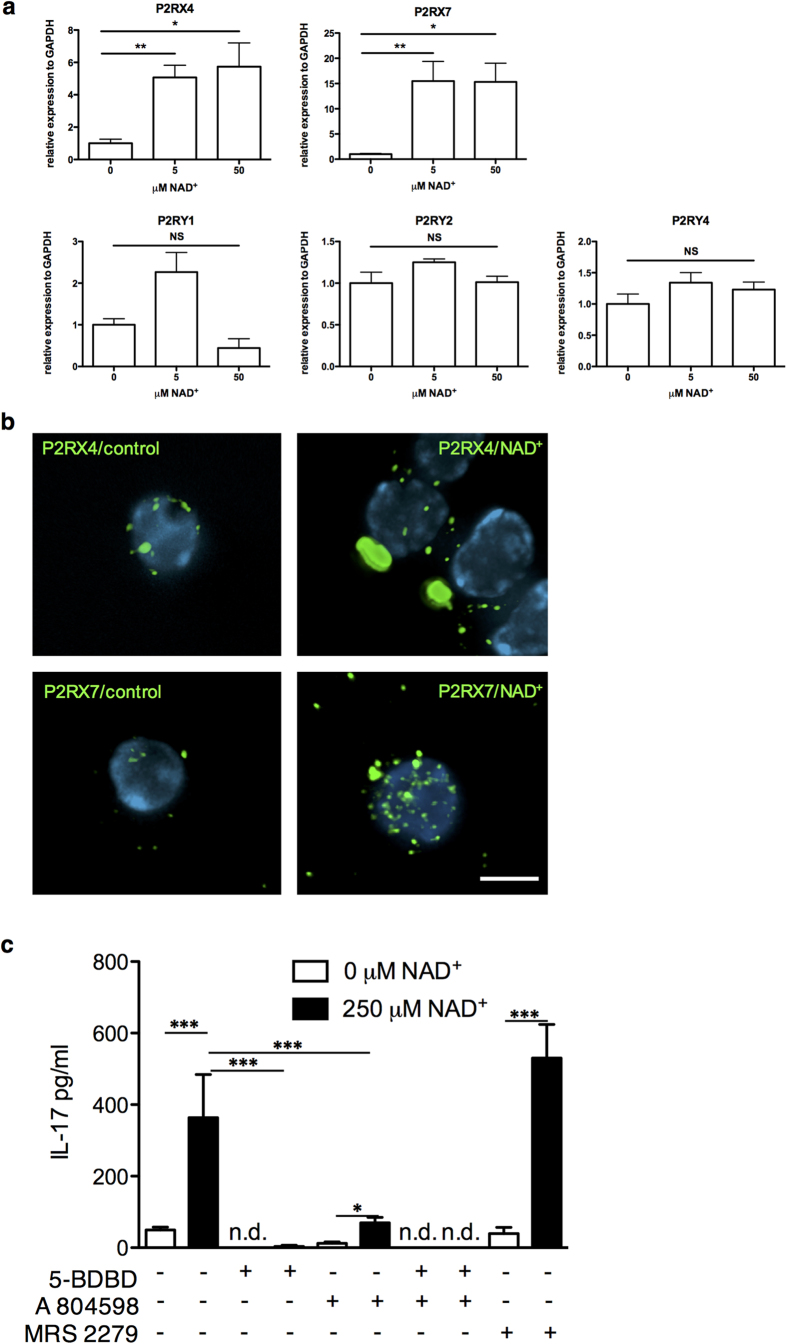
NAD^+^ signals through P2RX4 and P2RX7 receptors. CD4^+^ CD25^+^ Tregs were cultured in the presence of α-CD3, α-CD28, and IL-2 with increasing concentrations of NAD^+^; after 24 hrs (**a**) mRNA levels for *P2RX4*, *P2RX7*, *P2RY1*, *P2RY2*, and *P2RY4* were measured by real-time PCR (*n* = 6 per group). (**b**) Freshly isolated T cells were cultured for 24 hrs in presence of vehicle alone (right column) or 50 μM NAD^+^ (left column). Cells were collected and stained at 4 °C for either P2RX4 (top row) or P2RX7 (bottom row) without prior fixation or permeabilization. Stacks of 20, 2-channel images were acquired under each condition; resulting stacks were de-convolved and re-constituted for further analysis. Results show that treatment of T cells with NAD^+^ increased the cell surface expression levels of both receptors and in the case of P2RX4, NAD^+^ promotes capping-like distribution pattern. (**c**) Tregs were cultured as described above with or without NAD^+^ (250μM) and with or without MRS 2279 (a selective antagonist of P2RY1), 5-BDBD (a selective antagonist of P2RX4) or A 804598 (a selective antagonist of P2RX7) and IL-17A secretion was measured by ELISA (*n* = 6 per group). Data derived from two independent experiments. Data represent mean ± s.d. *P < 0.05; **P < 0.01; ***P < 0.001. ANOVA tests were used to compare groups.

Several purinergic receptors including P2RX4, 7 and P2RY1, 2, 4 have been reported to regulate T cell activation and function^28^. More importantly, it has recently been shown that purinergic activation, in particular that of P2RX7 by ATP promotes the conversion of Tregs into Th17 cells in the presence of IL-6^29^. It has also been shown that both ATP as well as NAD^+^ can activate purinergic receptors via the ART2.2 pathway^30^. Moreover, P2RX7 has a higher affinity to NAD^+^ when compared to ATP^31^,^32^. Thus, we tested whether purinergic signaling of NAD^+^ promoted the conversion of Tregs into Th17 cells. We therefore stimulated Tregs with anti-CD3/anti-CD28 in the presence of NAD^+^ and measured mRNA levels for *P2RX4*, *7* and *P2RY1*, *2*, *4* by real-time PCR at 24 hrs. Our findings indicated an up-regulation of *P2RX4* (>4 fold) and *P2RX7* (>15 fold) while *P2RY1*, *2* and *4* levels remained unchanged (Fig. 4a). Furthermore, immunofluorescence demonstrated that NAD^+^ increased the cell surface expression of *P2RX4* and *P2RX7* receptors resulting in their clustering (Fig. 4b, Supplementary Fig. 3; please see also our virtual reality quick time movies of 3-D reconstructed control and NAD^+^ treated T cells stained for P2RX4 and P2RX7 as Supplementary Movie S1A–D).

Next, we investigated whether the inhibition of P2RX4 and P2RX7 with highly selective antagonists blocked Treg conversion into Th17 by NAD^+^. When Tregs were cultured in the presence of 5-(3-Bromophenyl)-1,3-dihydro-2*H*-benzofuro[3,2-*e*]-1,4-diazepin-2-one (5-BDBD) and/or A804598, two selective antagonists of P2RX4^29^ or P2RX7^33^,^34^, respectively, we observed a dramatically reduced production of IL-17A that was complete when both selective antagonists were added (Fig. 4c). Of note, selective inhibition of P2RX4 resulted in a more robust blockade of IL-17 production when compared to P2RX7 (Fig. 4c). In contrast, a selective antagonist (MRS 2279) for P2RY1^35^ did not change IL-17A^+^ production by NAD^+^ (Fig. 4c). Collectively, these results suggest that the capacity of NAD^+^ to convert Tregs into Th17 cells is mediated through purinergic signaling.

### NAD^+^ prolongs allograft survival through a systemic increase of CD4^+^ IL-10^+^ producing cells

**Figure 5:**
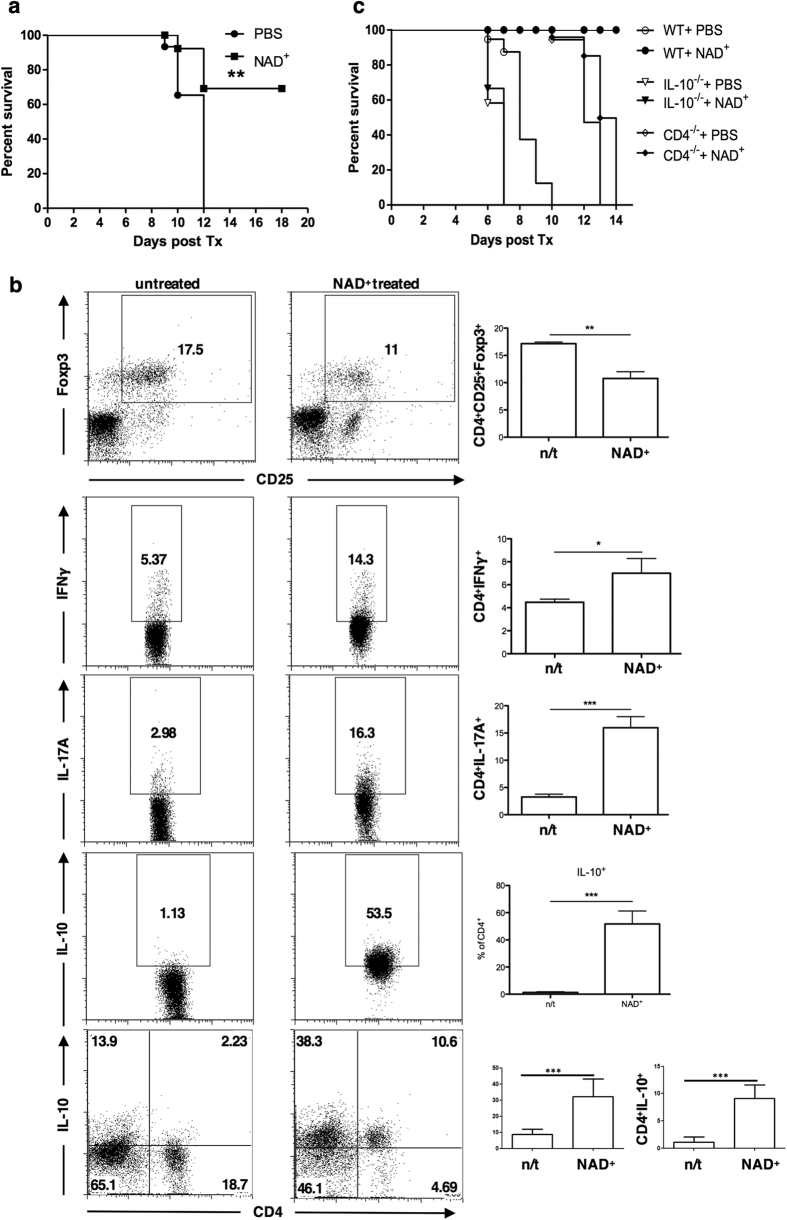
NAD^+^ promotes skin allograft survival through a systemic increase of IL-10. Fully MHC-mismatched C57BL/B6 tail skin allografts were transplanted onto DBA/2 mice that received daily doses of NAD^+^ (40 mg in 100 μl PBS) or control solution (PBS). (**a**) Skin graft survival was monitored (*n* = 6 per group) and (**b**) CD4^+^ T cells were isolated from spleens of wild type mice 8 days after transplantation and frequencies of IL-10^+^, IL-17A^+^, and IFN*γ*^+^ cells were analyzed by flow cytometry (*n* = 6 per group, representative plots shown). (c) Fully MHC-mismatched DBA tail skin allografts were transplanted onto *IL-10*^−/−^(C57BL/6 background), CD4^−/−^ (C57BL/6 background), (and wild type (WT) mice that received daily doses of NAD^+^ (40 mg in 100 μl PBS) or control solution (PBS) and skin graft survival was monitored (*n* = 6 per group). Data derived from two independent experiments. Data represent mean ± s.d. *P < 0.05; **P < 0.01; ***P < 0.001. Student’s *t*-test, ANOVA tests and Log-rank test were used to compare groups accordingly.

It is well established that Tregs promote allograft survival while Th17 cells enhance allograft rejection^36^,^37^. To further assess the unique therapeutic potential of NAD^+^ 24 and its role in Tregs fate we use of a fully MHC-mismatched mouse skin transplant model. C57BL/6 (H2^b^) tail skin allografts were transplanted onto DBA/2 (H2^d^) mice that received daily intraperitoneal injections of NAD^+^ or a placebo solution (PBS). Our results indicated that recipient mice treated daily with NAD^+^ exhibited a significantly prolonged allograft survival. Mean allograft survival in untreated recipient DBA mice was 10 days compared to >18 days in NAD^+^ treated recipient animals (Fig. 5a and Supplementary Fig. S4a). Next, we investigated how NAD^+^ alters recipient’s immune responses. Recipient mice were evaluated by day 8 and frequencies of CD4^+^ CD25^+^ Foxp3^+^ and CD4^+^ IL17A^+^ cells were assessed (Fig. 5b). We observed that recipient mice treated with NAD^+^ had reduced frequencies of CD4^+^ CD25^+^ Foxp3^+^ Tregs. More importantly, frequencies of CD4^+^ IL-17A^+^ and CD4^+^ IFNγ^+^ cells increased following NAD^+^ treatment (Fig. 5b). These findings were consistent with our recent study using an experimental autoimmune encephalomyelitis (EAE), the mouse model for human multiple sclerosis^24^. Moreover, our previous study^24^ suggests that the observed reduced frequencies of CD4^+^ CD25^+^ Foxp3^+^ Tregs after NAD^+^ administration^24^ and the increased frequency of Th17 cells^24^ may result of a conversion of Tregs into Th17 cells. Next, we studied mechanisms by which NAD^+^ could paradoxically deplete Tregs, increase Th1 and Th17 cells and at the same time promote allograft survival in a fully MHC-mismatched skin transplant mouse model.

The potent immunosuppressive capacity of IL-10 has been recognized before^38^. As shown in Figure 5b NAD^+^ treatment induced a systemic increase of IL-10 by CD4^−^ and CD4^+^ cells. More importantly, around 50% of CD4^+^ T cells in NAD^+^ treated mice were IL-10 producing cells (Fig. 5b) although CD4^+^ CD25^+^ Foxp3^+^ IL-10^+^ cells were reduced (Supplementary Fig. S5), suggesting that the systemic increased level of IL-10 was not mediated by CD4^+^ CD25^+^ Foxp3^+^ Tregs. Next, we investigated whether the prolonged allograft survival in NAD^+^ recipient mice was linked to a systemic increase of CD4^+^ IL-10^+^ cells. WT, *CD4*^−/−^ and *IL-10*^−/−^ mice (on a C57BL6/ background) received DBA skin transplants and were treated with NAD^+^. Of note, treatment with NAD^+^ in *CD4*^−/−^ and *IL-10*^−/−^ mice abolished a prolonged allograft survival (Fig. 5c and Supplementary Fig. S4b,c). Moreover, *IL-10*^−/−^ mice treated with PBS or NAD^+^ demonstrated a more rapid allograft rejection.

**Figure 6:**
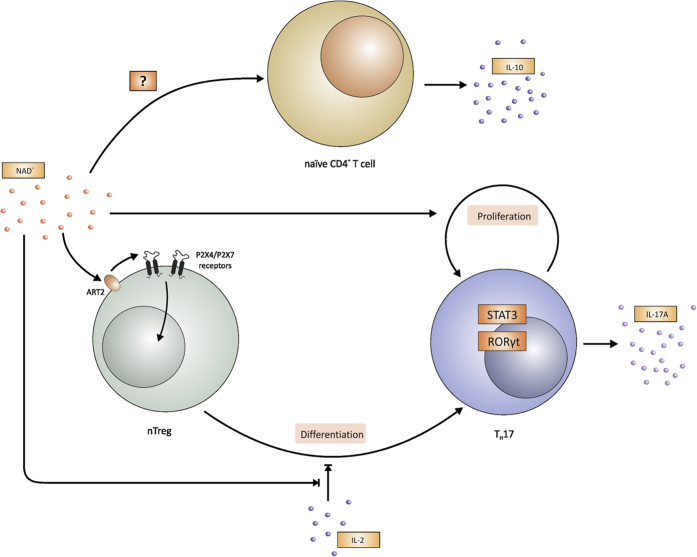
NAD^+^ regulates Treg conversion into Th17 cells and promotes homeostasis through IL-10. NAD^+^ promotes Tregs conversion into Th17 cells in absence of exogenous TGF-β and IL-6 cytokines after TCR engagement via purinergic receptors P2RX4 and P2RX7 and the transcription factors STAT3 and ROR*γ*t. Th17 cells differentiate in the presence of IL-2 and proliferate in absence of IL-23 cytokine. In addition, NAD^+^ promotes homeostasis via a robust systemic IL-10 originating from CD4^+^ T helper cells.

In summary, our results underscore the novel and unique therapeutic potential of NAD^+^ that mediates an impressive allograft survival through a systemic increase of IL-10 cytokine operating through a novel signaling pathway that does not dependent on CD4^+^ CD25^+^ Foxp3^+^ Tregs (Fig. 6).

## Discussion

It is well known that Th17 cell differentiation requires the addition of exogenous cytokines including TGF-β, IL-6 or IL-21, while IL-23 is required for their maintenance. In contrast, IL-2 has been shown to inhibit Th17 cell development. We have shown that NAD^+^ is able to induce a significant increase of IL-17 production in the absence of Th17 polarizing conditions and in the presence of high doses of IL-2. Moreover, NAD^+^ had the capacity to induce Th17 proliferation in the absence of IL-23. Overall our study demonstrated the ability of NAD^+^ to convert Tregs into IL-17A producing cells independently of the cytokine environment.

Of particular relevance, we have detected a novel mechanism by which NAD^+^ regulates the fate of CD4^+^ CD25^+^ Foxp3^+^ T cells. It has been shown that IL-2 is required for Treg maintenance and CD25 expression^39^. CD25 has been shown to be of critical importance for Treg survival and function. Furthermore, levels of CD25 have been reported as decisive for the differentiation of CD4^+^ Foxp3^+^ Tregs into Th17 cells^11^. The capacity of NAD^+^ to modify the binding of IL-2 to CD25 that impacts CD4^+^ CD25^+^ Foxp3^+^ T cell survival has been demonstrated recently^16^. Our study demonstrated that NAD^+^ induces the loss of CD25 expression, even in presence of IL-2 that has previously shown to up-regulate CD25^39^. Moreover, we were able to show that only CD4^+^ CD25^Low^Foxp3^+^ Tregs were able to produce IL-17A. These findings were consistent with a recent study showing that CD4^+^ CD25^Low^Foxp3^+^ but not CD4^+^ CD25^High^Foxp3^+^ Tregs were able to convert into Th17 cells^11^.

The conversion into IL-17A producing cells was associated with a dramatic change in the transcriptional signature profile documented by increased expression levels of *STAT3* and *ROR*γ*t*, two master regulators of Th17 development. Of note, using Tregs that are deficient for the transcription factor *STAT3*^−/−^, did not completely abolish the conversion of CD4^+^ CD25^+^ Foxp3^+^ Tregs into IL-17A^+^ producing cells, suggesting that additional mechanisms may be operative. In addition, changes in the transcriptional signature profile were also associated with modifications in the cytokine expression profile: mRNA and protein levels of IL-10, as well as mRNA levels of TFG-β, two cytokines produced by Tregs were reduced while IL-17A increased in a dose dependent manner. Of note, NAD^+^ impacted Th2 and Th1 cytokines only modestly.

Collectively, our results demonstrated that NAD^+^ promotes the conversion of CD4^+^ CD25^+^ Foxp3^+^ Tregs into CD4^+^ IL-17A^+^ T cells. The capacity of NAD^+^ to convert Tregs into IL-17A producing cells may explain increased numbers of CD4^+^ IL17A^+^ T cells observed in mice treated with NAD^+^. However, we cannot rule out that the increased Th17 response observed after NAD^+^ treatment may result from conventional CD4^+^ T cells as well.

Furthermore, it has been shown that both, ATP and NAD^+^ activate P2RX7 at very low concentrations^31^,^32^. In contrast to NAD^+^, ATP requires the presence of IL-6^29^ to convert Tregs into IL-17A producing cells suggesting that the higher affinity of NAD^+^ to purinergic receptors may induce a more robust response and/or activate additional signaling pathways. In addition, blocking P2RX4 and P2RX7 reduced Treg conversion into IL-17 producing cells, suggesting that these receptors may play differential roles in NAD^+^ signaling mechanisms and Treg conversion.

The *in vivo* and potentially clinical relevance of our findings has finally been demonstrated in a skin transplant mouse model. Recipient animals treated daily with NAD^+^ had a dramatically increased allograft survival when compared to controls. It is well established that Tregs play a key role in immunoregulation and T cell homeostasis. Moreover, it has been shown that Tregs promote allograft survival while IL-17A enhances allograft rejection. Interestingly, despite reduced numbers of CD4^+^ CD25^+^ Foxp3^+^ Tregs and despite the augmented Th17 response, NAD^+^ was able to promote a robust prolongation of allograft survival. On a mechanistic level, we found that NAD^+^ prolonged allograft survival by promoting a robust systemic IL-10 production. We have subsequently demonstrated the critical impact of IL-10 in communicating the immunoregulatory properties by NAD^+^ in *IL-10*^−/−^ mice. When *IL-10*^−/−^ mice were treated with NAD^+^, allograft survival was shorter than that observed in WT mice. These results were consistent with our previous study exploring the immunregulatory properties of NAD^+^ in the context of autoimmune disease^24^ and other reports indicating that IL-10 promotes allograft survival^38^. More importantly, decreased frequencies and reduced IL-10 expression of Tregs in our transplant model suggest that the increased frequency of CD4^+^ IL-10^+^ producing cells may not originate from Tregs but rather from conventional CD4^+^ T cells. Interestingly, we did not observe differences in transplant survival in *CD4*^−/−^ mice treated with PBS or NAD^+^ indicating that CD4^+^ T cells play a central role in NAD^+^-mediated allograft survival. Additional explorations on the impact of NAD^+^ on other key immune cells such as CD8^+^ T, dendritic and B cells will require exploration.

In summary, our study demonstrates that NAD^+^ modifies systemic immune responses in alloimmunity emphasizing on unique and novel immunoregulatory properties in addition to therapeutic applications of immunodeficient patients with *Foxp3* gene mutations.

## Materials and Methods

### Animals

Six to eight week old C57BL/6 (B6, H2^b^) and DBA/2 (H2^d^) mice were purchased from Charles River Laboratories (Wilmington, MA). *STAT3*^−/−^ (B6.129S1-*Stat3*^tm1Xyfu^/J) *CD4*^−/−^ (B6.129S2-Cd4^tm1Mak^/J), *IL-10*^−/−^ (B6.129P2-*IL10*^tmCgn^/J) and EGFP-FOXP3 transgenic mice were purchased from Jackson Laboratory. Animal use and care were in accordance with National Institutes of Health and Institutional Animal Care and Use Committee guidelines.

### Isolation of regulatory T cells

Single-cell leukocyte suspensions were obtained from spleens of wild type and EGFP-FOXP3 transgenic mice. CD4^+^ CD25^+^ FOXP3^+^ cells from EGFP-FOXP3 mice were cell sorted. For wild type mice, depletion of non-CD4^+^ T cells was performed using biotin-conjugated monoclonal anti-mouse antibodies against CD8α, CD11b, CD45R, CD49b, Ter-119 and anti-biotin magnetic beads (Miltenyi Biotec, Bergisch Gladbach, Germany). Cells were further sorted using α-CD25-PE and α-PE magnetic beads (Miltenyi Biotec). Purities of regulatory T cells after isolation were >98% (Supplementary Fig. S1a).

### Functional *in vitro* Treg cell assays

Isolated murine Treg cells were cultivated in 48-well flat bottom plates (2.5 × 10^4^ cells per well) in 0.5ml of complete media in presence of 10 μg/ml plate-bound anti-mouse α-CD3 (17A2) and 2 μg/ml soluble α-CD28 (37.51) in addition to 50 ng/ml recombinant mouse IL-2 (all eBioscience, San Diego, CA). NAD^+^ (Sigma-Aldrich) was added at 0, 5, 50or 250 μM and 5-BDBD, A804598 or MRS2279 (Tocris Bioscience, UK) was used where indicated to block P2RX4, P2RX7 or P2RY1 receptors, respectively. Cells were cultured for 24, 48 or 96 hrs and analyzed by flow cytometry. Supernatants were collected after 96 hrs and cytokine production was analyzed by ELISA.

### Flow cytometry

Fluorescently labeled anti-mouse α-CD4 (GK1.5), α-CD25 (PC61), α-CD11c (HL3), α-IFNγ (XMG1.2) and unlabeled α-CD16/CD32 (2.4G2) antibodies were obtained from BD Biosciences (San Jose, CA). Fluorescently labeled anti-mouse α-Foxp3 (FJK-16s), α-IL-17A (eBio17B7), α-IL-10 (JES5-16E3) were obtained from eBioscience. Fluorescently labeled anti-mouse α-CD4, α-CD44, α-CD62L were all obtained from eBioscience.

Intracellular staining for Foxp3, IL-17A, and IL-10 was performed according to manufacturers’ protocols. Splenocytes were re-stimulated in complete media (HL-1 media containing 10% FCS, 1% L-Glutamine, 1% Penicillin/Streptomycin; all Bio Whittaker, Walkersville, MD) for 4 hours at 37 °C with ionomycin (500 ng/ml) and phorbol 12-myristate 13-acetate (50 ng/ml, both Sigma-Aldrich, St. Louis, MO). Brefelding A (BD Biosciences) was added at a concentration of 0.67 μl/ml. Cells were fixed and permeabilized using Cytofix/Cytoperm solution (BD Biosciences) or Foxp3 fixation/permeabilization solution (eBioscience), respectively. Apoptosis staining with fluorescently labeled Annexin V (BD Bioscience) and proliferation assay with Carboxyfluorescein diacetate succinimidyl ester (Invitrogen, Carlsbad, CA) were both performed according to manufacturers’ protocols using commercial kits. To set the gates, flow cytometry dot plots were based on comparison with isotype controls, fluorescence minus one, permeabilized and unpermeabilized unstained cells.

Flow cytometry measurements of single-cell suspensions were performed on a FACSCalibur using standard procedures and data were analyzed using FlowJo software (Tree Star, Ashland, OR).

### ELISA

Mouse IL-4, IL-6, IL-10, IL-17A, IFNγ were measured using commercial kits (eBioscience). Briefly, ELISA plates were coated with 100 μl of anti-cytokine capture antibody at 4 °C overnight. Plates were then washed x5 with 0.05% PBS-Tween (PBST) and coated for 1 hr with blocking buffer provided by the manufacturer. Samples or standards were added in triplicates (100 μl/well) and incubated at 4 °C overnight. Wells were washed x5 with PBST and incubated with 100 μl of anti-cytokine detection antibody at 4 °C overnight. Wells were then washed x5 with PBST and incubated with 100 μl of avidin-HRP at room temperature for 30 min. Thereafter, wells were washed x7 with PBST and incubated with 100 μl/well of a substrate. The reaction was stopped after 15 min with 1M H_2_SO_4_ and absorbance was measured using a multiplate microplate fluorescence reader (Synergy HT, Biotekat) at 405 nm.

### RNA extraction and quantitative PCR

RNA extraction from isolated Treg cells after cultivation was performed using the RNAqueous extraction kit according to the manufacturer’s protocols (Applied Biosystems, Carlsbad, CA). Briefly, Treg cells were homogenized in lysis buffer (total volume of 0.5 ml) and passed through a column. After successive washes, RNA was eluted and reverse transcription was performed using i-Script cDNA synthesis kit (Bio-Rad Laboratories, Hercules, CA). PCR reactions were performed with Taqman primers and probes from Applied Biosystems. The housekeeping gene GAPDH was used as control. Relative gene expression was determined as described previously^39^.

### Microscopy, Deconvolution, 3D Reconstruction

Cultured T cells were stained on ice with 2 μg/ml of either control, anti-P2RX4 or anti-P2RX7 in presence of 2% IgG-free BSA (Life Technology) for 20 min, washed and incubated in the presence of the 0.01% Hoescht 33342 with relevant Alexa 488 secondary antibody as described previously^41^ (Alomone labs). After staining, cells were washed three times, mounted in fluorescence mounting media (DakoCytomation, Carpinteria, CA), and imaged using an Olympus BX62 motorized microscope fitted with a cooled Hamamatsu Orca AGCCD camera. The microscope, filters, and camera were controlled by Slide Book 5.0 (3I) Acquired Z-stacks were further processed using the deconvolution module of Volocity 5.0 (Improvision, Waltham, MA) followed by 3-D surface rendering reconstruction using maximum intensity projection algorithms. ImagePro Plus 7.0 software (Mediac Cybernetics) was used for quantification of positive area and signal intensity^30^.

### Skin transplantation model

Full-thickness tail skin grafts (~1 cm^2^) were procured from C57BL/6 mice and engrafted onto the dorsolateral thoracic wall of DBA/2 recipient mice using interrupted 5-0 Vicryl sutures. 40 mg of NAD^+^ in 100 μl PBS, or 100 μl PBS alone were injected intraperitoneally and grafts were covered with gauze and adhesive bandage for 5 days. Graft survival was then monitored daily and rejection was defined as graft necrosis of 100%. Two investigators blinded for the particular experimental groups assessed graft survival independently. Eight days after transplantation, single-cell leukocyte suspensions were obtained from spleens procured from recipient mice to perform re-stimulation (with PMA and Ionomycin for 2 hrs) and staining for surface and intracellular antigens as described above.

### Statistical analysis

Values and error bars represent mean ± s.d. Data were analyzed using GraphPad Prism (GraphPad Software, San Diego, CA). Log-rank test was used to compare survival curves. Unpaired two-tailed Student’s t-test was used when comparing two groups. To compensate for multiple comparison errors ANOVA tests were used. A *p* value < 0.05 was considered statistically significant.

### Ethics Statement

This study was approved by the Institutional Animal Care and Use Committee (IACUC). All the experiments described here were performed in accordance with the approved guidelines of the National Institutes of Health (NIH) and the IACUC.

## Additional Information

**How to cite this article**: Elkhal, A. *et al.* NAD^+^ regulates Treg cell fate and promotes allograft survival via a systemic IL-10 production that is CD4^+^ CD25^+^ Foxp3^+^ T cells independent. *Sci. Rep.* **6**, 22325; doi: 10.1038/srep22325 (2016).

## Supplementary Material

Supplementary Information

Supplementary Movie s1a

Supplementary Movie s1b

Supplementary Movie s1c

Supplementary Movie s1d
