# Supplementary material for: NAD+ regulates Treg cell fate and promotes allograft survival via a systemic IL-10 production that is CD4+ CD25+ Foxp3+ T cells independent
Source: Sci Rep. 2016 Mar 1;6:22325. doi: 10.1038/srep22325 (PMC4772111; doi:10.1038/srep22325)
Supplement: Supplementary Information [file srep22325-s1.pdf]

SUPPLEMENTAL MATERIALS

NAD^+ regulates Treg cell fate and promotes allograft survival via a systemic IL-10 production that is $CD4^+CD25^+Foxp3^+$ T cells independent

Abdallah Elkhal[1,*,†], Hector Rodriguez Cetina Biefer[1,2,†], Timm Heinbokel[1,3], Hirofumi Uehara[1], Markus Quante[1], Midas Seyda[1], Jeroen M. Schuitenmaker[1] Felix Krenzien[1], Virginia Camacho[4], Miguel A. de la Fuente[5], Ionita Ghiran[6] and Stefan G. Tullius[1,*]

[1]Division of Transplant Surgery and Transplantation Surgery Research Laboratory, Brigham and Women's Hospital, Harvard Medical School, Boston-02115, MA; [2]Clinic for Cardiovascular Surgery, University Hospital Zurich, Zurich-8006, Switzerland; [3]Institute of Medical Immunology, Charité – Universitätsmedizin Berlin, Berlin-10117, Germany; [4]Flow Cytometry Core Facility, Beth Israel Deaconess Medical Center, Harvard Stem Cell Institute, Boston, Massachusetts 02115, USA. [5]Instituto de Biología y Genética Molecular, University of Valladolid, Valladolid-47003, Spain; [6]Department of Medicine, Beth Israel Deaconess Medical Center, Harvard Medical School, Boston, Ma. 02115, USA

[†]A.E. and H.R.C.B. contributed equally to this work and are co-first authors.

[*]Correspondence and requests for materials should be addressed to:

Abdallah Elkhal, Ph. D.
Transplant Surgery Research Laboratory
Brigham and Women's Hospital
221 Longwood Avenue
Boston, MA 02115, USA
Email: aelkhal@partners.org
Phone: 001-617-732-5659

Stefan G. Tullius, MD, PhD
Division of Transplant Surgery
Brigham and Women's Hospital
75 Francis Street
Boston, Ma. 02115, USA
Email: stullius@partners.org
Phone: 001-732 6446

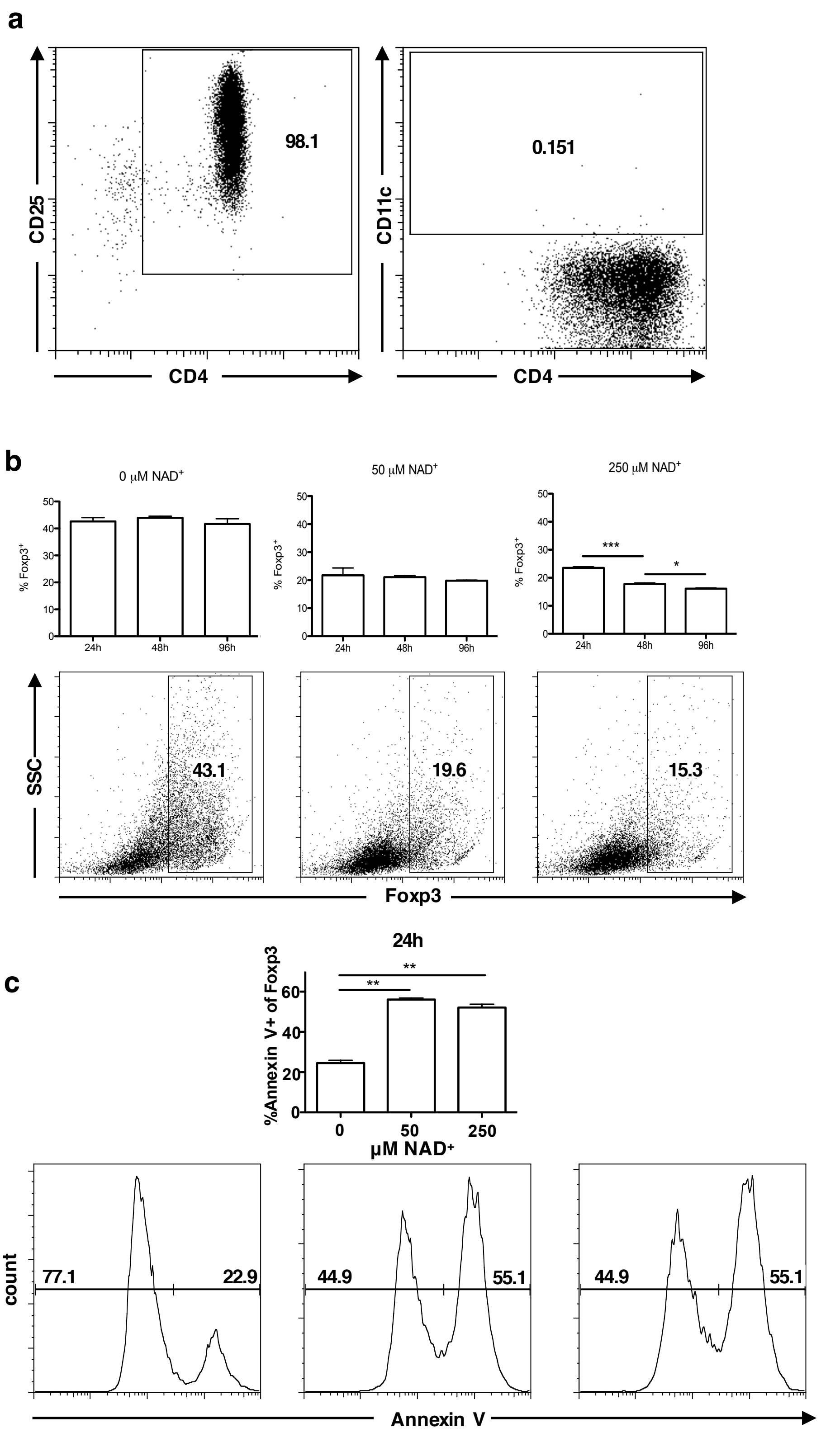

Supplementary Figure S1: NAD+ induces Treg apoptosis and loss of Foxp3 expression in vitro. (a) Purities of $CD4^{+}CD25^{+}$ Tregs after isolation from spleens of DBA mice were >98%, containing no contaminating $CD11c^{+}$ cells (plots shown are representative for three independent experiments). (b) Frequencies of Tregs that were cultured in presence of α-CD3, α-CD28, and IL-2 and increasing concentrations of NAD^{+} after 24hrs, 48hrs and 96hrs (n=6 per group, representative plots shown). (c) Percentages of Annexin V^{+} cells among $CD4^{+}CD25^{+}Foxp3^{+}$ Tregs after 24hrs of culture in presence of α-CD3, α-CD28, and IL-2 and increasing concentrations of NAD^{+} (n=6 per group, representative plots shown). *, $P<0.05$; **, $P<0.01$; ***, $P<0.001$. Student's *t*-test was used to compare between groups.

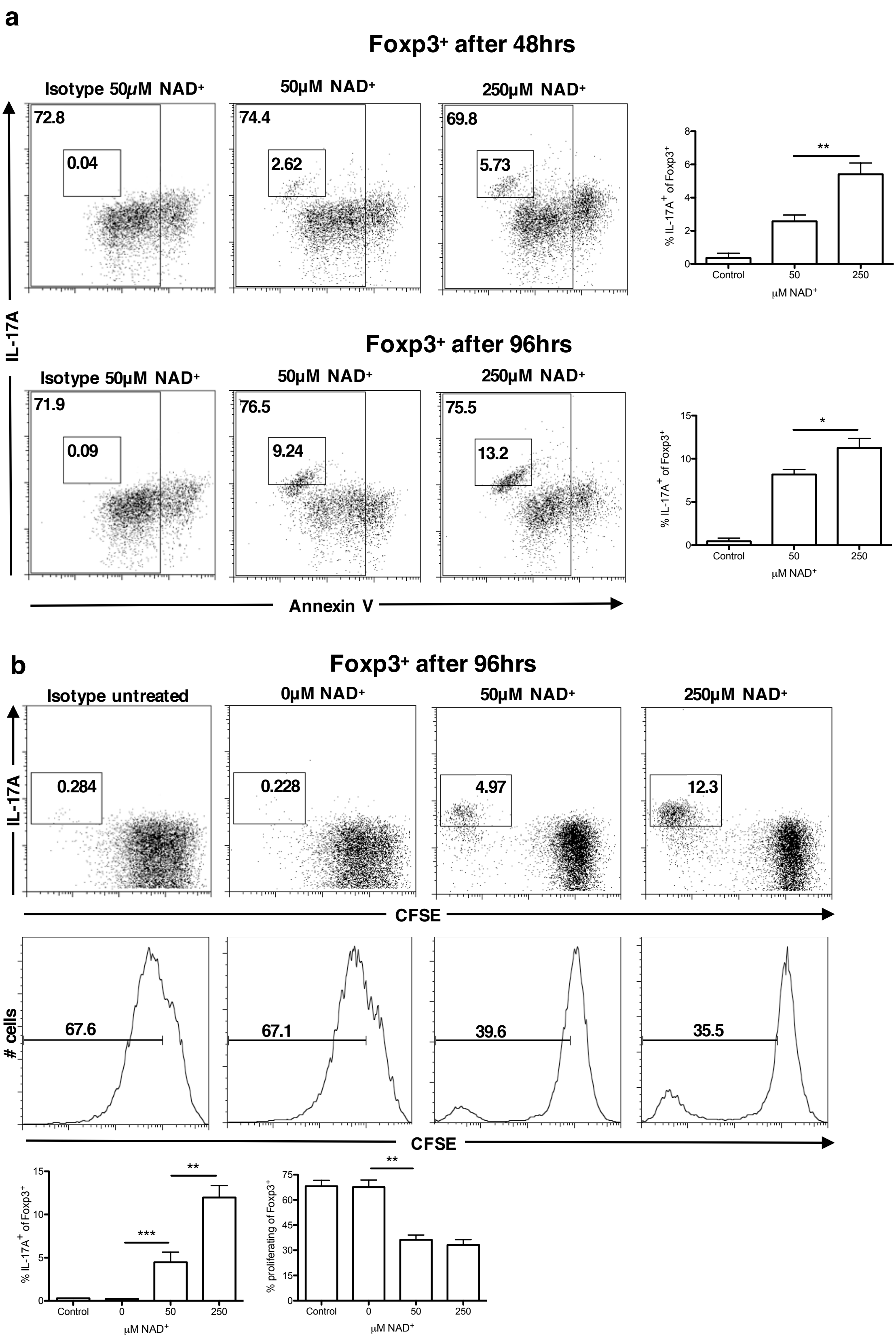

Supplementary Figure S2: Tregs converted into IL-17A producing cells remain resistant to apoptosis and proliferate in presence of NAD$^+$ without exogenous IL-23 cytokine. CD4$^+$CD25$^+$Foxp3$^+$ cells were cultured in presence of α-CD3, α-CD28, and IL-2 with increasing concentrations of NAD$^+$ and (a) apoptosis of CD4$^+$CD25$^+$Foxp3$^+$IL-17A$^+$ cells was assessed with Annexin V after 48hrs and 96hrs in a dose-dependent manner (*n*=6 per group; data derived from two independent experiments; representative plots shown). (b) Proliferation was assessed with CFSE in a dose-dependent manner (*n*=6 per group; data derived from two independent experiments; representative plots shown).

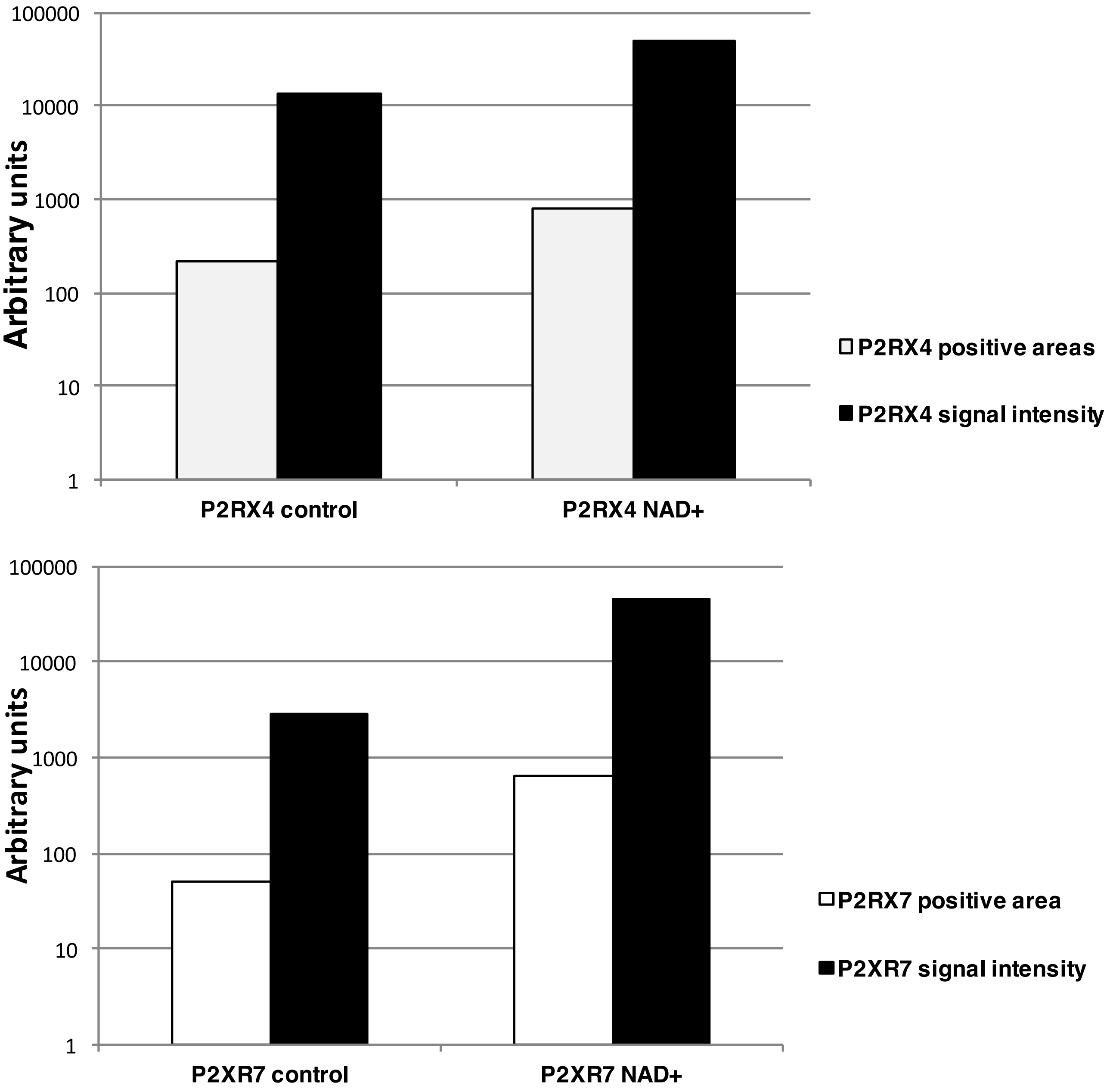

Supplementary Figure S3: NAD+ increases P2RX4 and P2RX7cell surface expression. Isolated T cells were cultured for 24hrs in presence of vehicle alone (right column) or 50μM NAD+ (left column). Cells were collected and stained at 4°C for either P2RX4 (top row) or P2RX7 (bottom row) without prior fixation or permeabilization. Stacks of 20, 2-channel images were acquired under each condition; resulting stacks were de-convolved and re-constituted for further analysis. Results shown Quantification of positive area as well as signal intensity.

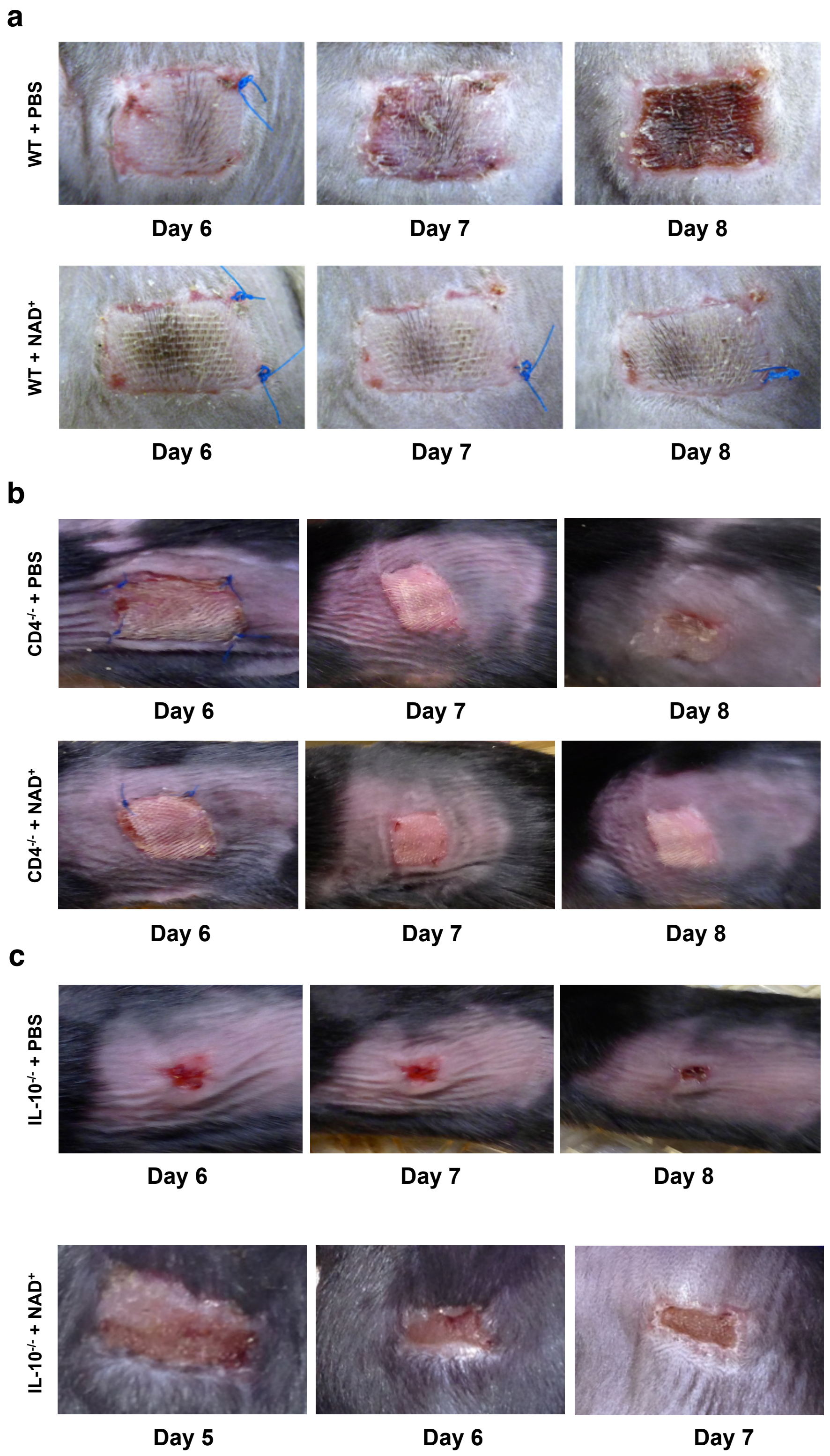

Supplementary Figure S4: NAD+ promotes allograft survival. Photos of skin grafts from day 6 to day 13 after transplantation of mice treated with NAD+ or with a placebo solution. Fully MHC-mismatched tail skin allografts from C57BL/6 mice were transplanted onto DBA/2 recipient mice that received (A) control solution (PBS) or daily doses of NAD+ (40 mg). (B) Fully MHC-mismatched DBA tail skin allografts were transplanted onto *CD4-/-* mice (C57BL/6 background) that were treated daily with NAD+ (40 mg). (C) Fully MHC-mismatched DBA tail skin allografts were transplanted onto *IL-10-/-* mice (C57BL/6 background) that were treated daily with NAD+ (40 mg). Photos of skin grafts were taken daily from day 6 to day 13. For *IL-10-/-* mice photos of skin grafts were taken daily from day 5 to day 7. *n*=5 mice per group; representative images are shown.

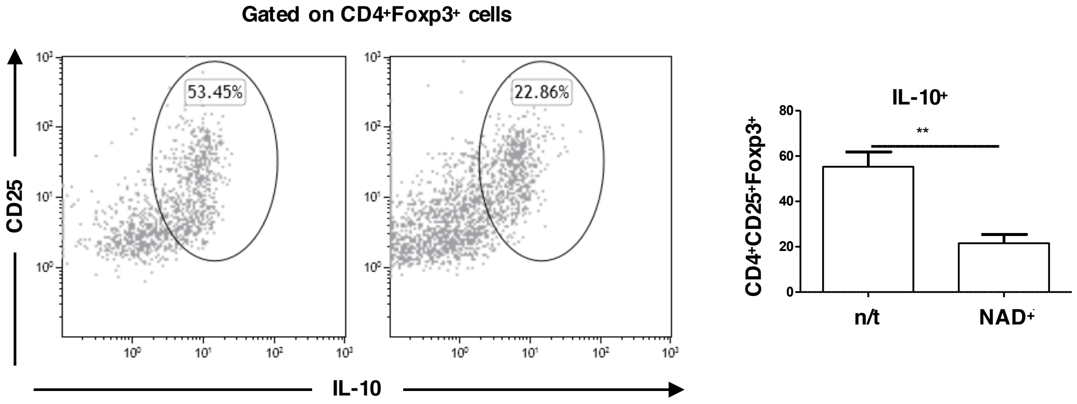

Supplementary Figure S5: Increased systemic production of IL-10 is not mediated through Tregs. Fully MHC-mismatched C57BL/B6 tail skin allografts were transplanted onto DBA/2 mice that received daily doses of NAD^+ (40 mg in 100µl PBS) or control solution (PBS). $CD4^+$ T cells were isolated from spleens 8 days after transplantation and gated on $CD4^+Foxp3^+$ cells and frequencies of $IL\text{-}10^+CD25^+$ were analyzed by flow cytometry (*n*=6 per group, representative plots shown).

Supplementary Movie S1: NAD^+ promotes cell surface expression of P2RX4 and P2RX7 receptors on Tregs. P2RX4 expression by Tregs in presence of (a) PBS solution or (b) 50µM of NAD^+ after 24hrs of culture. P2XR7 expression by Tregs in presence of (c) PBS solution or (d) 50µM of NAD^+ after 24hrs of culture.
